# Cell senescence alters responses of porcine trabecular meshwork cells to shear stress

**DOI:** 10.3389/fcell.2022.1083130

**Published:** 2022-11-21

**Authors:** Ruotian Du, Dongyan Li, Meng Zhu, Lisha Zheng, Keli Ren, Dong Han, Long Li, Jing Ji, Yubo Fan

**Affiliations:** ^1^ Key Laboratory of Biomechanics and Mechanobiology of Ministry of Education, Beijing Advanced Innovation Center for Biomedical Engineering, School of Biological Science and Medical Engineering, Beihang University, Beijing, China; ^2^ Lab for Biological Imaging and Nanomedicine, National Center for Nanoscience and Technology, Beijing, China; ^3^ State Key Laboratory of Nonlinear Mechanics and Beijing Key Laboratory of Engineered Construction and Mechanobiology, Institute of Mechanics, Chinese Academy of Sciences, Beijing, China

**Keywords:** shear stress, senescence, trabecular meshwork cell, glaucoma, mechanobiology

## Abstract

Mechanical microenvironment and cellular senescence of trabecular meshwork cells (TMCs) are suspected to play a vital role in primary open-angle glaucoma pathogenesis. However, central questions remain about the effect of shear stress on TMCs and how aging affects this process. We have investigated the effect of shear stress on the biomechanical properties and extracellular matrix regulation of normal and senescent TMCs. We found a more significant promotion of Fctin formation, a more obvious realignment of F-actin fibers, and a more remarkable increase in the stiffness of normal cells in response to the shear stress, in comparison with that of senescent cells. Further, as compared to normal cells, senescent cells show a reduced extracellular matrix turnover after shear stress stimulation, which might be attributed to the different phosphorylation levels of the extracellular signal-regulated kinase. Our results suggest that TMCs are able to sense and respond to the shear stress and cellular senescence undermines the mechanobiological response, which may lead to progressive failure of cellular TM function with age.

## Introduction

As the second major cause of blindness globally (Bourne, 2006; Cook and Foster, 2012), glaucoma is a group of eye diseases that lead to optic nerve damage and consequent irreversible visual loss (Tian et al., 2017). Glaucoma is often classified into several types, among which primary open-angle glaucoma (POAG) is one of the most common ones (Weinreb and Khaw, 2004). Nowadays, POAG affects approximately 57.5 million people worldwide (Wiggs and Pasquale, 2017; Allison et al., 2020), and it is predicted that approximately 111.8 million people will suffer from glaucoma by 2040 (Tham et al., 2014).

Although the molecular mechanism of glaucoma pathogenesis is poorly understood, elevated intraocular pressure (IOP) resulting from increased resistance to aqueous humor outflow in the TM conventional outflow pathway is considered one of the main risk factors for POAG (Gabelt and Kaufman, 2005; Mcmonnies, 2017; Buffault et al., 2020; Li and Song, 2020). Located in the anterior chamber angle of the eye, TM is a mechanosensitive tissue that mediates 80–90% of aqueous outflow (Yuan et al., 2016; Yarishkin et al., 2021). TMCs are able to adjust the aqueous outflow facility through remodeling the actin cytoskeleton (Clark et al., 2005; Rao et al., 2005; Rao et al., 2017), or changing extracellular matrix (ECM) turnover and subsequent ECM replacement rates by modulating matrix metalloproteinases (MMPs) activity (Bradley et al., 2001; Keller et al., 2009; Pattabiraman and Rao, 2010; Vranka and Acott, 2017). Impaired regulation of these cellular functions leads to IOP dysregulation and has been advocated as a pathogenic factor of POAG (Last et al., 2011; Vahabikashi et al., 2019). Although the underlying mechanisms remain elusive, it has been reported that the extracellular signal-regulated kinase (ERK) pathway is involved in regulating the production of MMPs in TM (Alexander and Acott, 2003). Studies indicate that the ERK pathway can affect the secretion of MMPs in TMCs, which may lead to an aberrant accumulation of ECM and consequently elevated IOP that eventually develops glaucoma (Shearer and Crosson, 2001; Conley et al., 2004).

The bulk flow of aqueous humor driven by IOP imposes shear stress on the conventional outflow pathway (Wudunn, 2009; Yarishkin et al., 2021). This shear stress is predicted to be in the range of 2–25 dyn/cm^2^, which could be higher due to the elevated IOP in glaucoma (Ethier et al., 2004). Existing studies suggested that the TMCs could respond to shear stress imposed by aqueous humor flow (Carreon et al., 2017), thus providing a means of regulatory feedback to control IOP (Johnstone, 2004; Yarishkin et al., 2021). Recently, it has been proven that the shear stress-induced change of TMCs may be involved in the increase in outflow resistance in glaucoma. For example, Patel et al. found that impaired TRPV4-eNOS signaling activated by increased fluid shear stress in TMCs contributes to elevated IOP in glaucoma (Patel et al., 2021). Meanwhile, Yarishkin et al. reported that the shear stress could activate Piezo1, leading to an increased number of focal cell-matrix contacts of human TMC, a determinant of mechanically induced aqueous humor outflow which in turn contributes to TM stiffness (Yarishkin et al., 2021). But the effects of shear stress on the important cellular functions of TMC, such as cytoskeleton remodeling, cell migration, and MMP production, remain unknown so far.

The risk of developing POAG clearly increases with age (Yoshida et al., 2001; Friedman et al., 2004; Cook and Foster, 2012). Aging is a process associated with the accumulation of damages that brings about a progressive decline in cellular and physiological function, which can attenuate the cellular capacity to feel and respond to stress and then increase the risk of degenerative diseases. According to the oxidative stress theory, the accumulation of negative effects induced by reactive oxygen species (ROS) results in progressive loss of functions in aging (Liguori et al., 2018). The TM is the most sensitive tissue to oxidative damage in the anterior chamber (Izzotti et al., 2009). Senescence of the TMCs is assumed as a major risk factor in the development or progression of POAG (Liton et al., 2008; Liton et al., 2009). Numerous studies have demonstrated that cell senescence could alter the morphology (Saccà et al., 2016), cytoskeleton (Zhou et al., 1999), phenotype (Liton et al., 2005), and functions (Alvarado et al., 2005; Zhao et al., 2016) of the TMCs. However, how cell senescence influences the TMCs’ response to shear stress is barely known.

Here, we investigated the effects of senescence on the responses of porcine trabecular meshwork (PTM) cells to shear stress. Our results showed that the mechanotransduction of PTM cells could be altered by cell senescence. A more remarkable realignment of F-actin fibers, a more significant promotion of Fctin formation, and a greater increase in cell stiffness in response to the shear stress were observed in normal PTM cells compared with senescent PTM cells. Shear stress enhanced the capability of cell migration of normal PTM cells whereas decreased that of senescent PTM cells. Moreover, senescent PTM cells exhibited altered changes in ECM turnover-related protein after the shear stress stimulation in comparison with normal PTM cells, which may be associated with the difference in phosphorylation levels of ERK. Our findings indicate that the cell senescence compromises the physiological responses of PTM cells to shear stress.

## Materials and methods

### Cell culture of primary porcine trabecular meshwork cells

Primary cultures of PTM cells were prepared from porcine eyes obtained from the local abattoir within less than 4 h postmortem. Briefly, the TM was dissected from surrounding tissue as previously described (Obazawa et al., 2004; Liton et al., 2008). The tissue was then placed in collagen I-coated 35-mm dishes and cultivated in a TM culture medium which consists of low-glucose Dulbecco’s modified Eagle’s medium (DMEM; Hyclone, United States) supplemented with l-glutamine and 110 mg/L sodium pyruvate, 100 mM nonessential amino acids, 100 U/mL penicillin, 100 mg/ml streptomycin sulfate and 20% fetal bovine serum (FBS) (Li et al., 2007). All the reagents were obtained from Invitrogen (Carlsbad, CA). After one passage, serum was reduced to 10% for routine cultivation. Cells were maintained and propagated at 37 °C in humidified air with 5% CO_2_. When the cells reached confluency, they were subcultivated 1:3. The passages three to four of the PTM cell were used in our study. More than 20 different porcine cell lines were studied. For each experiment, we used at least three different cell lines. The PTM cells used in this study were characterized as previously described (Polansky et al., 2000; Snider et al., 2015; Keller et al., 2018). Briefly, cultured cells at passage three were grown to confluence and then treated with 100 nM dexamethasone for 7 days before the myocilin mRNA expression level was examined. Also, cells at passage three were carefully seeded on the prepared slide and the expression of fibronectin (FN) and laminin (LN) was evaluated by immunochemical staining.

### Experimental model of senescence in PTM cells

PTM cells were subjected to normobaric hyperoxia conditions as previously described (Saretzki et al., 1998; Liton et al., 2008). Confluent cultures of PTM cells at passage three were grown for 2 weeks at 40% O_2_ and 5% CO_2_ in a triple-gas incubator (China Innovation Instrument, Ningbo, China). Control cultures were grown under 5% CO_2_ and atmospheric oxygen concentration. The senescence β-galactosidase staining kit (Beyotime, Shanghai, China) was used according to the manufacturer’s protocol to stain senescent PTM cells. The staining was visualized with a Nikon Eclipse inverted microscope system. The percentage of senescent cells was calculated as the number of cells that contained the blue β-galactosidase staining divided by the total number of cells in the field of view.

### Cell cycle assay

Cell cycle was determined by flow cytometry as previously described (Zheng et al., 2016). Cells were incubated with 20 μg/ml DNase-free RNase A (Beyotime, Shanghai, China) at 37°C, washed with PBS (phosphate buffer saline) and stained with propidium iodide (PI) (Beyotime, Shanghai, China). Cell cycle was analyzed using BD CellQuest Pro software by FACSCalibur™ (BD, Franklin Lakes, NJ, United States).

### Application of shear stress

A parallel plate flow chamber was utilized to produce shear stress as described previously (Zheng et al., 2016; Huang et al., 2019). The PTM cells cultured on glass slides were placed in the flow block that can provide controlled shear stress *τ* = 6*Qμ*/(*wh* ^2^), where the shear stress (*τ*) depended on the flow rate (*Q*), the viscosity of the culture media (*μ*), as well as the width (*w*) and height 8) of the flow channel. The whole device was incubated at 37°C in a humidified atmosphere of 5% CO_2_ in the air. A steady laminar shear flow of 25 dyn/cm^2^ was loaded.

### Fluorescent labeling of actin structures and image analysis

Cells were fixed in 4% paraformaldehyde, then were permeabilized with 0.1% Triton X-100 in PBS and blocked in 1% bovine serum albumin. Cells were incubated in Texas red isothioc2yanate-conjugated phalloidin (Invitrogen, Carlsbad, CA) for 30 min to stain the F-actin filaments. Then the cells were incubated with DAPI (Sigma, St. Louis, MO, United States) for 5 min to label cell nuclear. The fluorescent images were taken under a confocal microscope (TCS-SP5; Leica, Solms, Germany). The angle of cell is defined as the angle between the main axis of the cell and the flow direction. The cell angles and average optical intensity of F-actin were calculated with ImageJ software (NIH Image, Bethesda, MD).

### Cell stiffness measurement

Cell stiffness was determined as described previously (Ding et al., 2015; Sancho et al., 2017; Gu et al., 2018) using the atomic force microscope (AFM, Agilent 5,500, United States) equipped with an inverted fluorescence microscope (Nikon TE 2000U). Acquisition of F-D curves was carried out on Picoview SPM System (Agilent 5,500, United States). We employed a very low loading rate of 0.25 μm/s, and an indentation depth of 300 nm. The spherical probe was applied here to determine the overall stiffness of each cell. The calibrated spring constant was given as 0.08 N/m. The probe tip was a SiO_2_ sphere with a diameter of 11.41 μm. Cells were selected randomly and each of them was compressed three times. More than 10 cells were measured for each treatment condition and each experiment was repeated three times. The F-D curves were fitted by the Hertz model. A MATLAB program was used to process all the data.

### Migration assay

The migration of PTM cells was measured with a transwell migration apparatus as described previously (Hogg et al., 2000; Kim, 2016). Briefly, cells were trypsinized and resuspended at a density of 6×10^5^ cells/ml in serum-free media. Then, the PTM cells were added into the upper wells of a transwell chamber (Corning, United States). Culture media with 10% FBS were added into the lower wells. After incubating for 24 h, cells were fixed and stained with DAPI. Migrated cells attached to the bottom of the filter were counted under a fluorescent microscope.

### Total RNA extraction and reverse transcription-polymerase chain reaction analysis

After removing the culture medium, PTM cells were immediately immersed in RNAlater™ (Qiagen, Valencia, CA) to preserve RNA integrity. Total RNA was isolated from PTM cultures using an RNase kit (Qiagen) according to the manufacturer’s protocol and was treated with DNase. RNA yields were determined using Ribogreen fluorescent dye (Molecular Probes). First-strand cDNA was synthesized from 0.5 µg total RNA by reverse transcription using an oligo dT primer and Superscript II reverse transcriptase (Invitrogen) according to the manufacturer’s instructions. Reverse transcription-polymerase chain reaction (RT-PCR) analyses were performed using the PCR parameters shown in Table 1. Glyceraldehyde 3-phosphate dehydrogenase (GAPDH) or β-actin was used as an internal standard of mRNA expression. The sequences of the primers used for the amplifications are listed in Table 2.

**TABLE 1:** Parameters used for RT-PCR.

	Temperature and time		
Gene	34–36 cycles		
	Denaturation	Annealing	Extension
MYOC	30 s at 94°C	30 s at 58°C	1 min at 72°C
GAPDH	30 s at 94°C	30 s at 56°C	1 min at 72°C
MMP-1	30 s at 94°C	30 s at 57°C	1 min at 72°C
MMP-2	30 s at 94°C	30 s at 57°C	1 min at 72°C
TIMP-1	30 s at 94°C	30 s at 55°C	1 min at 72°C
TIMP-2	30 s at 94°C	30 s at 55°C	1 min at 72°C
COLA-1	30 s at 94°C	30 s at 57°C	1 min at 72°C
COLA-4	30 s at 94°C	30 s at 54°C	1 min at 72°C
β-actin	30 s at 94°C	30 s at 64°C	1 min at 72°C

**TABLE 2:** Sequence of the primers used for RT-PCR.

Gene	Forward	Reverse
MYOC	AGG​GAA​GTT​TCT​AAA​TGG​AAT​GTG​G	CCA​GTG​ATT​GTC​TCG​GCT​GT
GAPDH	CAG​CAA​TGC​CTC​CTG​TAC​CA	GAT​GCC​GAA​GTT​GTC​ATG​GA
MMP-1	CAC​ACA​CCT​GAC​CTA​CAG​GAT​T	TGG​GAC​AGC​TGA​ACA​TCA​CC
MMP-2	GAC​GTG​ACC​CCA​TTA​CGG​TT	CTT​CAC​ACG​CAC​CAC​TTG​TC
TIMP-1	CAC​CTG​CAG​TTT​TGT​GGC​TC	GGG​ATG​GAT​GTG​CAG​GGA​AA
TIMP-2	CGT​TTT​GCA​ATG​CAG​ACG​TAG	CGC​GTG​ATC​TTG​CAC​TCA​CA
COLA-1	AGA​CAT​CCC​ACC​AGT​CAC​CT	TCA​CGT​CAT​CGC​ACA​ACA​CA
COLA-4	GTG​CAT​GCG​GAG​AAC​ATG​AC	AGG​GTG​TGT​TAG​TTA​CGC​GG
β-actin	AAG​ATC​AAG​ATC​ATC​GCG​CCT​CCA	TGG​AAT​GCA​ACT​AAC​AGT​CCG​CCT

### Western blot analysis

Cell lysates were prepared using RIPA solution, and protein concentration was determined with a BCA protein determination kit (Ythxbio, China). Equal amounts of protein samples (25 μg) were separated by SDS-PAGE (10.0% acrylamide gel slabs) and then transferred to PVDF membranes (Bio-Rad). The PVDF membranes were blocked with 5% BSA and incubated overnight with anti-GAPDH antibody (Beyotime, China), ERK antibody (Santa Cruz, CA), or p-ERK antibody (Santa Cruz, CA), followed by incubation with secondary antibodies conjugated to peroxidase. GAPDH was used as a loading control.

### Statistic analysis

These experiments were repeated at least three times independently with different cell lines, as described above. Data were represented as the mean ± SD and were analyzed by one-way analysis of variance (one-way ANOVA). Data analysis was performed with GraphPad Prism7 (GraphPad Software Inc., United States) and SPSS 19.0 (SPSS Inc., United States). Differences were considered statistically significant at *p* < 0.05.

## Experimental results

### Characterization of PTM cells

Morphologically, confluent cultures of cells exhibited the typical morphology of PTM cells, i.e., long shuttle in shape (Supplementary Figure S1B) (Mao et al., 2013; Stamer and Clark, 2017). Measured by immunohistochemical staining, the cells in this study expressed TM cells biomarker FN and LN (Supplementary Figure S1C) (Khaw et al., 1994; Ge et al., 2016; Wang et al., 2017; Huang et al., 2022). Because the neighboring cells do not respond as robustly, the induced myocilin expression in response to DEX is widely accepted as a gold standard in TM cell characterization (Polansky et al., 2000; Snider et al., 2015; Gu et al., 2018). In this study, 100 nM DEX was added for 7 days to provoke robust myocilin production, as assessed by PCR **(** Supplementary Figure S1D, E **)**. By examining cell morphology, biomarkers, and myocilin induction, the identity of TM cells was established.

### Hyperoxia-induced cellular senescence model

**FIGURE 1:**
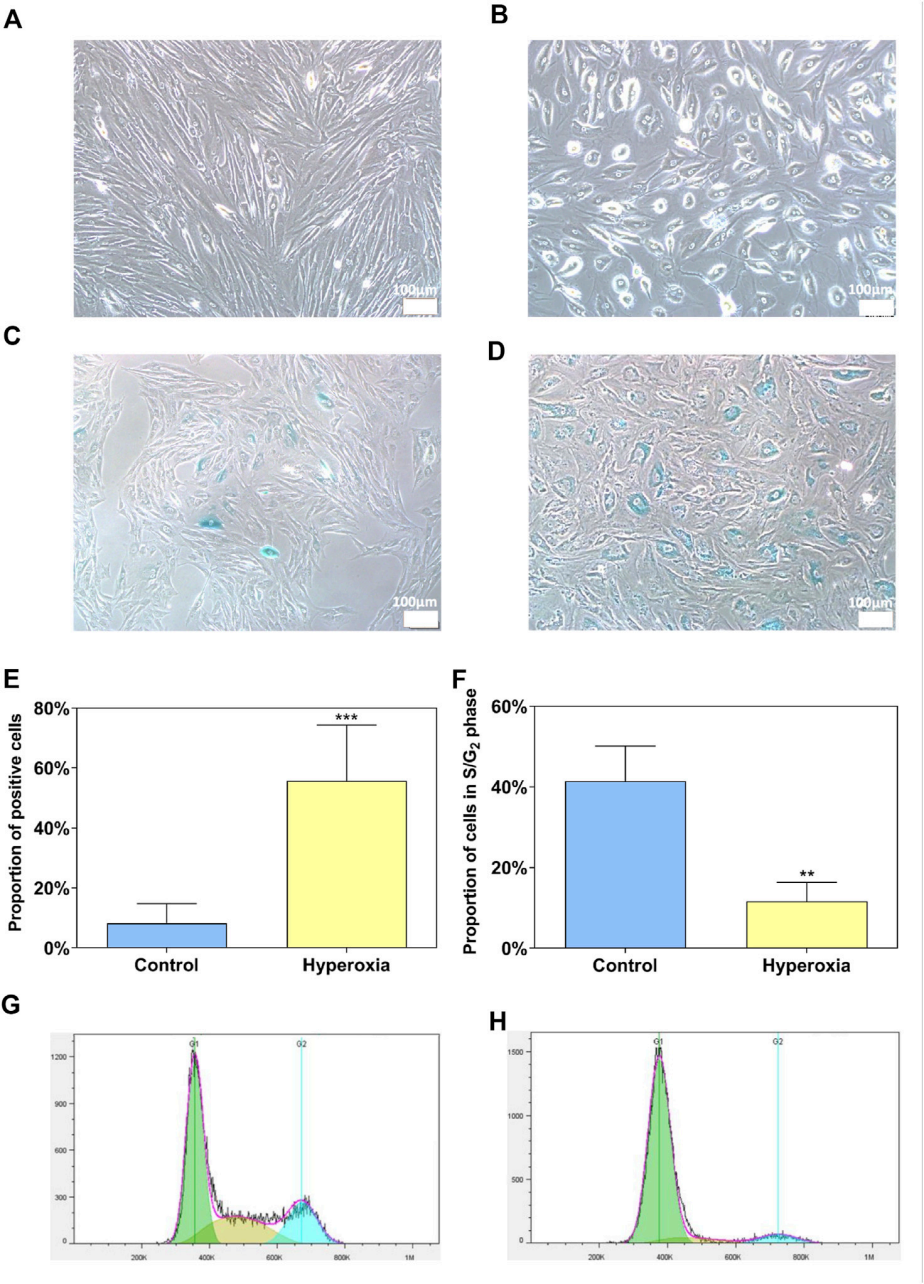
Hyperoxia as an experimental model of senescence for porcine trabecular meshwork (PTM) cells. Morphology of PTM cells grown for 2 weeks under control **(A)** or hyperoxic (40% O_2_) conditions **(B)**. β-galactosidase staining of PTM cells grown for 2 weeks under control **(C)** or hyperoxic (40% O_2_) conditions **(D)**. PTM cells grown under the control conditions exhibited negligible staining for senescence marker β-galactosidase, whereas cells exposed to hyperoxia stained positive for this marker **(E)** (***p < 0.001). The proportion of cells in the S phase and G2 phase decreased after hyperoxia exposure compared with the control group **(F)** (**p < 0.01). Flow cytometry quantification of the cell cycle of PTM cells grown for 2 weeks under control **(G)** or hyperoxic (40% O_2_) conditions **(H)**.

In this study, we adopted the normobaric hyperoxia treatment to induce senescent cells (Gille and Joenje, 1992; Liton et al., 2008). We found that these cells grown in 40% O_2_ exhibited morphology with enlarged cell size (Figure 1B) compared to normal PTM morphology (Figure 1A). After 2-week exposure to hyperoxia, PTM cells stained positively for the cell senescence marker β-galactosidase (Figure 1D), whereas PTM cells in control have negligible staining for this maker (Figure 1C), as shown in Figure 1E. Further, flow cytometry results showed that the proportion of G_2_/M phase cells appeared to decrease after exposure to hyperoxia (Figure 1H) compared to the control group (Figure 1G). Quantitative results were shown in Figure 1F.

### Effect of senescence on cytoskeleton and cell stiffness of PTM cells in response to shear stress

**FIGURE 2:**
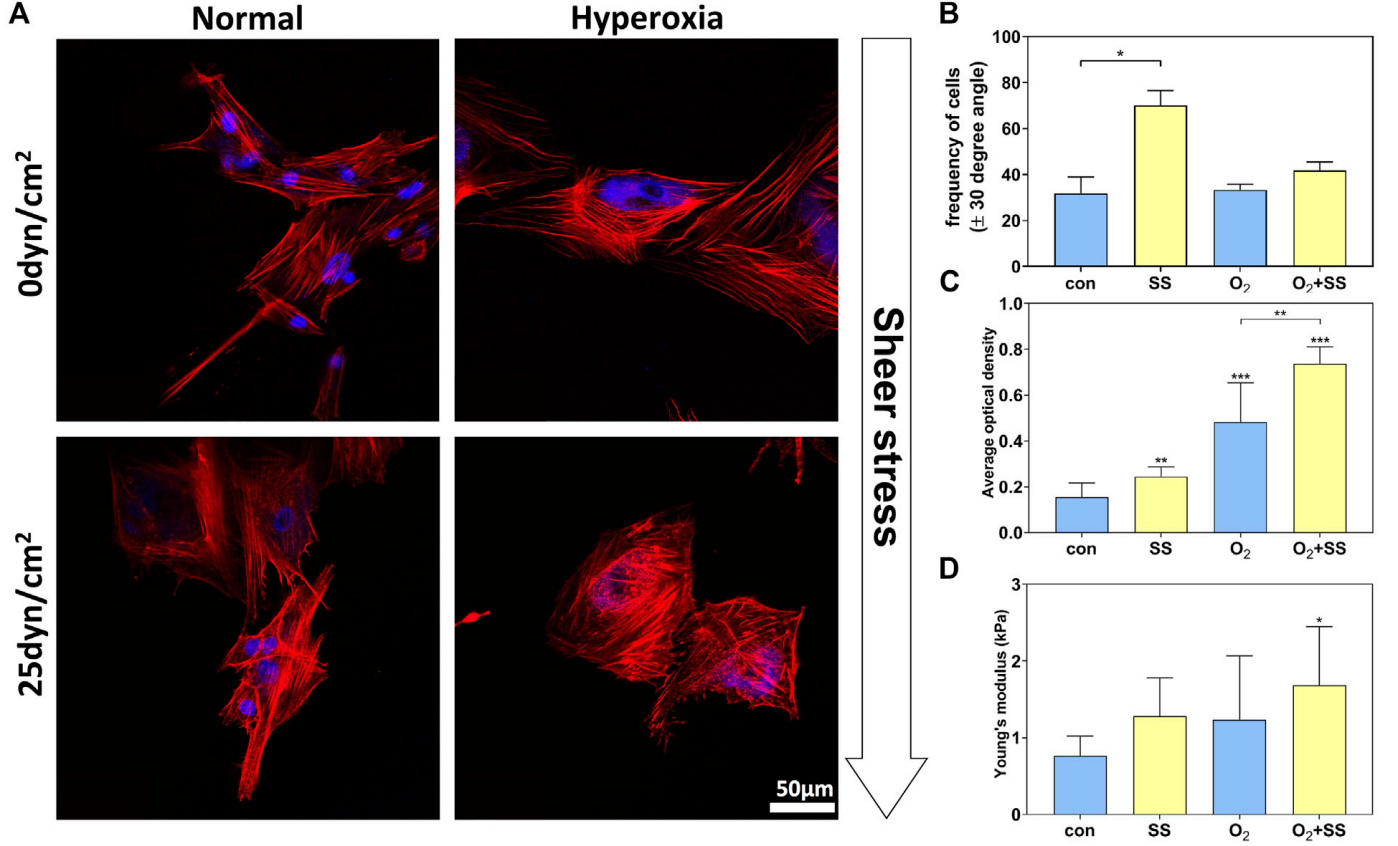
Effect of senescence on cytoskeleton and cell stiffness of PTM cells in response to shear stress. **(A)** Fluorescence images of normal and senescent PTM cells under static conditions (no shear stress) or subjected to shear stress of 25 dyn/cm^2^. Red: phalloidin, Blue: DAPI. **(B)** Alignment of the cytoskeleton of normal and senescent PTM cells under static conditions or subjected to shear stress (*p < 0.05). **(C)** F-actin content of normal and senescent PTM cells under static conditions or subjected to shear stress (**p < 0.01, ***p < 0.001). **(D)** Cell stiffness of normal and senescent PTM cells under static conditions or subjected to shear stress (*p < 0.05). SS stands for shear stress.

The quantified alignment of PTM cells was depicted as a proportion of cells within ±30° range with respect to the flow axis. For normal PTM cells, it was shown in Figures 2A,B that the percentage of cells aligned with the angle of orientation ranging from -30° to 30° with respect to the flow axis (0°) was significantly increased after 12-h exposure to shear stress of 25 dyn/cm^2^ compared with the static group (no shear stress). In contrast, for senescent PTM cells, the percentage of cells did not vary significantly after exposure to sheer stress compared with the static group (Figures 2A,B). These results suggested that, after exposure to shear stress of 25 dyn/cm^2^ for 12 h, normal PTM cells tended to orient in the direction of the flow, while senescent PTM cells did not respond in the same way.

As shown in Figure 2C, these senescent PTM cells demonstrated a significant increase in F-actin content compared with normal PTM cells. When exposing cells to shear stress, F-actin content was significantly improved in both normal and senescent PTM cells. Correspondingly, AFM results indicated that the senescence of PTM cells led to an increase in cell stiffness (Figure 2D). After being subjected to the shear stress, both normal and senescent PTM cells exhibited increased cell stiffness. Quantitatively, we observed an increase in stiffness by 67.44% and 36.14% for normal and senescent PTM cells, respectively, after exposure to the shear stress. As expected, simultaneously exposing PTM cells to hyperoxia and shear stress led to the most remarkable change in cell stiffness compared with the control (Figure 2D). Our findings are consistent with existing results which suggested that the cell stiffness is positively correlative to the F-actin content (Starodubtseva, 2011).

### Effect of senescence on cell migration of PTM cells in response to shear stress

**FIGURE 3:**
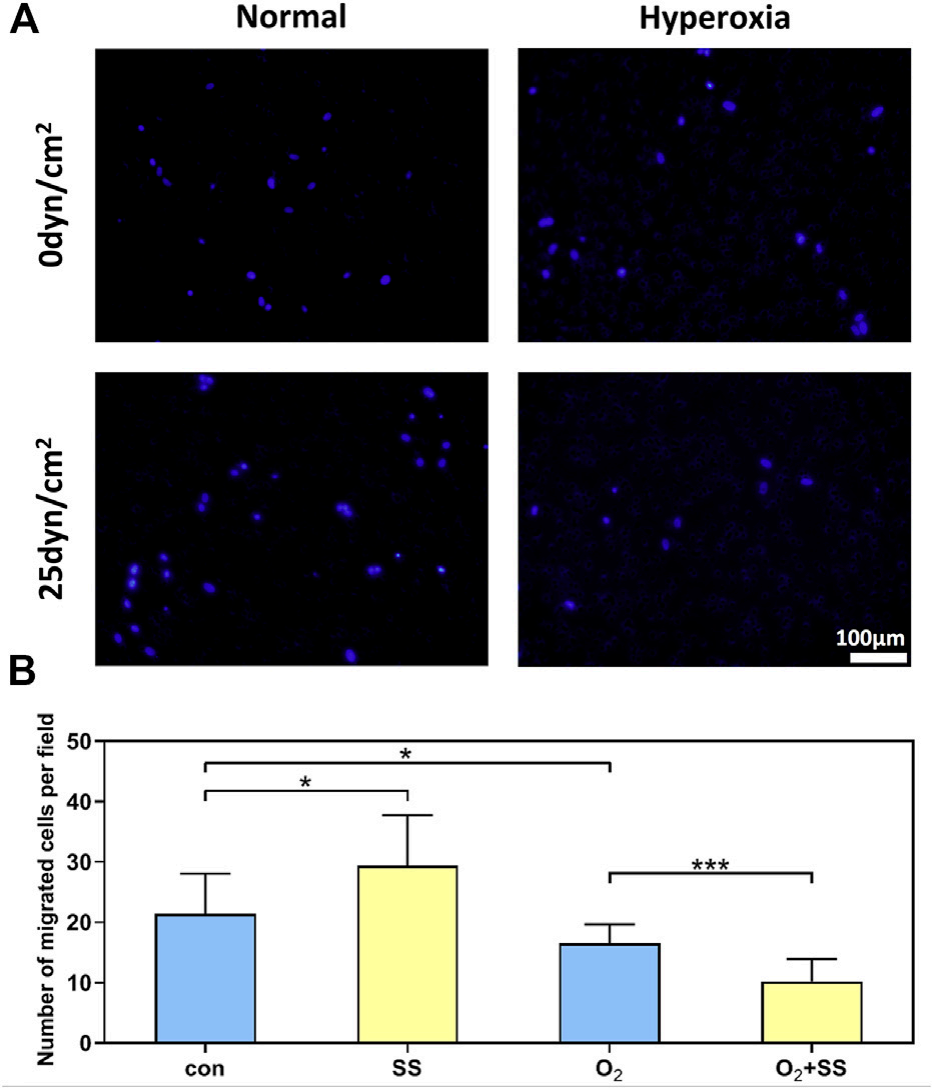
Effect of senescence on cell migration of PTM cells in response to shear stress. **(A)** Transwell migration assay for normal and senescent PTM cells under static conditions or subjected to shear stress **(B)** Migration activity of normal and senescent PTM cells under static conditions or subjected to shear stress (*p < 0.05, ***p < 0.001). SS stands for shear stress.

Transwell assays were performed to determine the migration ability of normal and senescent PTM cells subjected to the shear stress. We found that the senescent PTM cells had a significantly lower migration rate compared with normal PTM cells. After 12-h exposure to the shear stress, the migration ability of normal PTM cells significantly increased compared with the static group. However, the opposite was true for the senescent PTM cells (Figures 3A,B).

### Effect of senescence on MMP, tissue inhibitors of metalloproteinases (TIMPs) and collagen mRNA expression of PTM cells in response to shear stress

**FIGURE 4:**
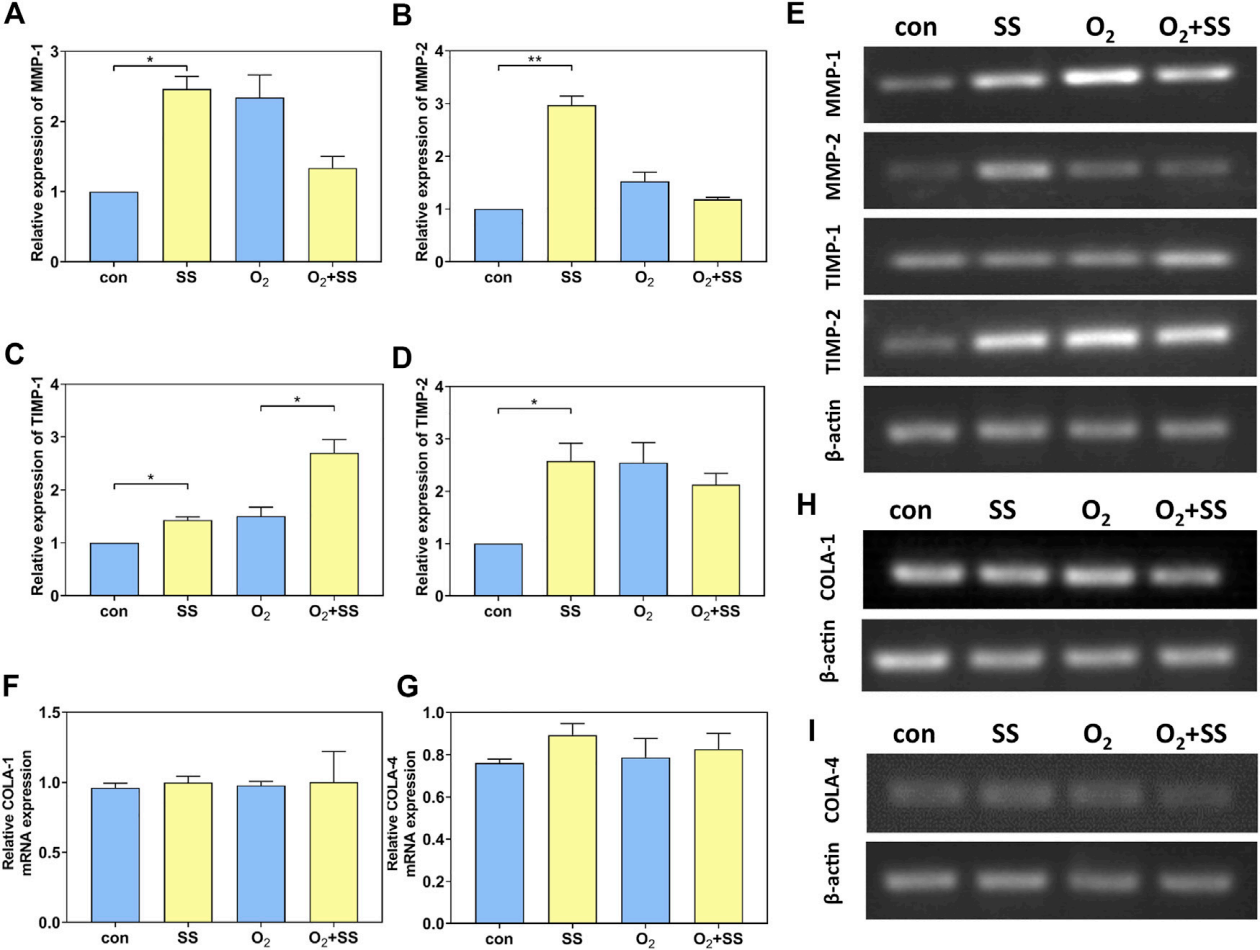
Effect of senescence on matrix metalloproteinases (MMP), tissue inhibitors of metalloproteinases (TIMP), and collagen mRNA expression of PTM cells in response to shear stress. **(A–E)** PCR quantification of MMP-1, MMP-2, TIMP-1 and TIMP-2 mRNA expression of normal and senescent PTM cells under static conditions or subjected to shear stress (*p < 0.05, **p < 0.01). **(F, H)** PCR quantification of collagen I (COLA-1) mRNA expression of normal and senescent PTM cells under static conditions or subjected to shear stress. **(G, I)** PCR quantification of collagen IV (COLA-4) mRNA expression of normal and senescent PTM cells under static conditions or subjected to shear stress. SS stands for shear stress.

Results from RT-PCR indicated that, for normal PTM cells, the mRNA expressions of MMP-1, MMP-2, TIMP-1, and TIMP-2 were significantly up-regulated after 12-h exposure to the shear stress compared to the static group. However, the mRNA expressions of these specific proteins, except TIMP-1, were significantly down-regulated for senescent PTM cells (Figures 4A–E). In addition, the mRNA expression of collagen I (COLA-1) and collagen IV (COLA-4) did not change for both normal and senescent PTM cells after exposure to the shear stress (Figures 4F–I).

### Effect of senescence on the ERK protein expression of PTM cells in response to shear stress

**FIGURE 5:**
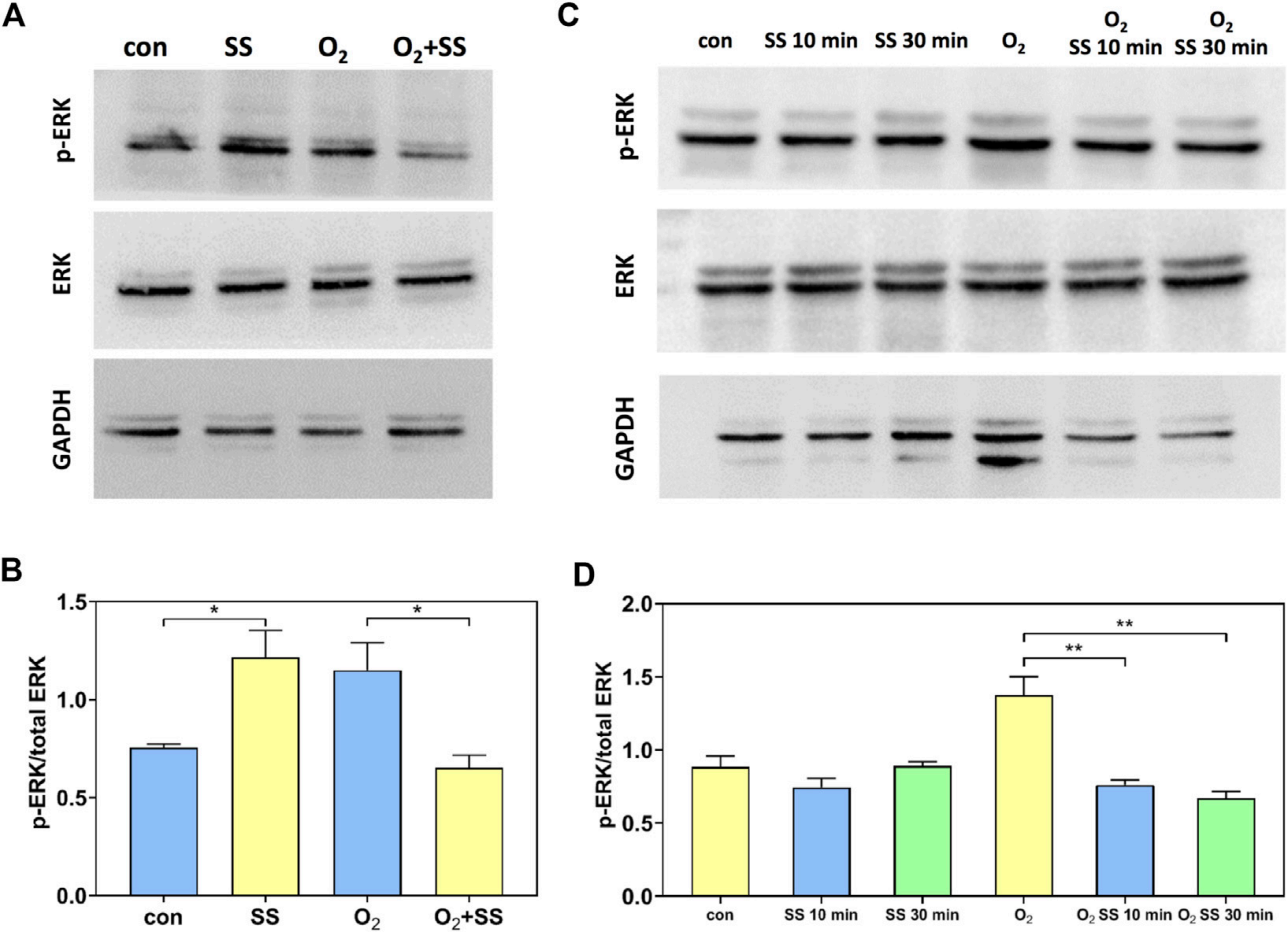
Effect of senescence on the extracellular signal-regulated kinase (ERK) and p-ERK expression of PTM cells in response to shear stress. **(A,B)** Western blot analysis of the p-ERK and ERK expression of normal and senescent PTM cells under static conditions or subjected to 12-h shear stress (*p < 0.05). **(C,D)** Western blot analysis of the p-ERK/total ERK protein expression of normal and senescent PTM cells under static conditions or subjected to 10-min and 30-min shear stress (**p < 0.01). SS stands for shear stress.

We also studied the expression and phosphorylation of ERK using Western blot analysis. For normal PTM cells, the p-ERK/total ERK ratio increased significantly after 12-h exposure to the shear stress compared with the static group. In contrast, a significant decrease in p-ERK/total ERK ratio was observed for senescent PTM cells in response to the 12-h exposure to the shear stress (Figures 5A,B). To further study the effect of senescence on the ERK expression and phosphorylation, we investigated the exposure time-dependence of normal and senescent PTM cells by considering short-term shear stress. As shown in Figures 5C,D, for normal PTM cells, the p-ERK/total ERK ratio remained roughly unchanged after exposure to the shear stress for 10 min and 30 min compared to the static group. However, the p-ERK/total ERK ratio decreased significantly to the value measured for 12 h after exposure to the shear stress even for 10 min for the case of senescent PTM cells (Figures 5C,D). These results indicated that the alteration in ERK phosphorylation in response to the shear stress occurred at a different time point in normal or senescent PTM cells.

## Conclusion and discussion

In this study, we investigated the effects of senescence on the responses of PTM cells to the shear stress. We found for the first time that mechanotransduction of PTM cells could be altered by cell senescence. Our studies addressed the possible correlation of the senescence-induced change in cytoskeletal rearrangement, F-actin remodeling, migration, and MMP/TIMP expression in response to shear stress with the pathogenesis of POAG.

It is commonly accepted that the TMCs can remodel the cytoskeleton in response to mechanical stress such as mechanical stretch or ECM stiffness in order to moderate their capability of draining aqueous humor (Starodubtseva, 2011; Li et al., 2022). In our study, following exposure to the shear stress of 25 dyn/cm^2^, normal PTM cells showed clearly a cytoskeletal rearrangement, i.e., the F-actin fibers tended to realign along the direction of the flow, in sharp contrast to the random cytoskeletal arrangement for cells grown under static conditions. The cytoskeletal network’s structure is essential for transmitting force stimulation and perceiving the mechanical microenvironment cues (Janmey and Mcculloch, 2007; Du et al., 2022). It may play a crucial role in several biological functions of TMCs, such as cell contraction, cell migration, and phagocytosis (Rottner et al., 2017; Mylvaganam et al., 2021). Hence, we propose the cytoskeletal rearrangement of TMCs as an important means for responding to the shear stress and regulating the aqueous humor outflow. Existing studies have proved that the shear stress can cause cytoskeletal arrangement for several different cell types (Galbraith et al., 1998; Kadi et al., 2007; Huang et al., 2010; Risca et al., 2012; Cheng et al., 2013; Molladavoodi et al., 2017; Son et al., 2020), and the changes in cytoskeleton and cellular functions after shear stress stimulation may be mediated by FAK (Girard and Nerem, 1995; Fabry et al., 2011; Cheng et al., 2013; Sun et al., 2018), ERK pathway (Fabry et al., 2011; Sun et al., 2018), Rho pathway (Tzima et al., 2002), and transient receptor potential melastatin type 7 (TRPM7) channel (Liu et al., 2015; Xiao et al., 2015). These uncovered mechanisms may also hold for the case of TMCs. An exciting result in our study was that the shear stress-induced reorganization of the cytoskeleton was weakened for the senescent PTM cells. Existing studies indicated that the cytoskeletal arrangement of TMCs in glaucomatous eyes of elderly human is more random and disordered than that in control eyes (Read et al., 2007; Huang et al., 2022). Moreover, glaucomatous TMCs derived from POAG donors are insensitive to shear stress (Patel et al., 2021). Therefore, we postulate that the impairment of rearrangement in senescent TMCs under mechanical stimulation may partly contribute to the aging-related glaucoma pathogenesis.

We further found that the F-actin content of normal PTM cells increased under shear stress stimulation. This finding indicated that not only the cytoskeletal organization but also the formation of F-actin could be actively changed after exposure to the shear stress. It has been revealed that the TMCs showed increased stress fibers in response to mechanical stimulation such as cyclic mechanical stretch (Duffy and O’reilly, 2018). For other cell types, the F-actin formation had also been shown to correlate closely with the shear stress (Okuyama et al., 1996; Schleicher et al., 2008; Mu et al., 2015), depending on the mode and intensity of shearing (Chen et al., 2004). The dynamic regulation of F-actin polymerization of TMCs in response to the shear stress may be significant for the maintenance of outflow resistance and IOP homeostasis. Interestingly, our results showed that the shear stress stimulation led to moderate but less significant increase in F-actin content in senescent PTM cells than that in normal PTM cells. This indicated that the cell senescence impaired the capability of dynamic regulation of F-actin polymerization. It is well recognized that the F-actin is a major determinant in maintaining the cellular elastic stiffness (Fallqvist et al., 2016), and the enhancement of cell stiffness is associated with F-actin formation (Sun et al., 2017). Here, we measured the stiffness of normal and senescent PTM cells after shear stress stimulation using AFM. Our results indicated that the senescent PTM cells with more F-actin exhibited higher cell stiffness (Morgan et al., 2015) and showed a less significant increase in cell stiffness after exposure to the shear stress. Altogether, cell senescence may impair the cytoskeleton formation in TMCs after shear stress stimulation and consequently affect the regulation of cell stiffness.

The cell migration, a highly dynamic process driven by the cytoskeleton (Lin et al., 2019), was also found to be affected by the shear stress and cell senescence. More specifically, the migration of normal PTM cells increased after shear stress stimulation, while the opposite is true for the senescent PTM cells. This may be attributed to the difference in the response of cytoskeleton to the shear stress for normal and senescent cells. The relationship between the migration of TMCs and elevated IOP is not clear so far. For example, Koga et al. suggest that inhibition of migration activities might be associated with decreased aqueous outflow (Koga et al., 2006). While, Igarashi et al. suppose that inhibition of cell migration or proliferation could benefit glaucoma treatment (Igarashi et al., 2021). Although the relationship between migration of TMCs and elevated IOP remains to be established, it is widely believed that migration activities of TMCs might be associated with aqueous outflow, suggesting migration as a potential therapeutic target in treating glaucoma. We propose that normal PTM cells can sense the shear stress induced by fluid flow and accordingly moderate their functions such as migration to regulate IOP. While these responses in senescent PTMs cells are negated, which might be associated with dysregulated aqueous humor outflow and IOP.

MMPs, as a family of zinc-dependent enzymes that are involved in the ECM degradation (Clark, 1998), have been proposed to play a vital role in regulating the ECM turnover in the TM and the IOP (Alexander et al., 1991). Among those enzyme family members, TMCs are known to express MMP-1, MMP-2, and their endogenous inhibitors TIMPs, which are important for the modulation of aqueous humor outflow facility by controlling ECM turnover, cell growth, and cell migration in the TM (Sivak and Fini, 2002; Pang et al., 2003; Brew and Nagase, 2010; Ramer and Hinz, 2010). It has been demonstrated that mechanical stimulation, e.g., strain and mechanical stretch, can influence the expression of MMPs in TMCs (Wudunn, 2001; Bradley et al., 2003). In our study, we found that the expression of MMP-1 and MMP-2 in normal PTM cells increased after shear stress stimulation, which was in contrast down-regulated in senescent PTM cells. This different response to the shear stress for normal and senescent PTM cells was also observed in the cell migration as discussed above. Another finding is that the level of MMPs and TIMPs in senescent cells are higher than those in normal cells at zero stress, suggesting a higher ECM turnover rate which is believed as a pathological change in glaucomatous eyes (Camras et al., 2012; Camras et al., 2014). We further evaluated the mRNA expression of COLA-1 and COLA-4, which are vital components of the ECM in TM (Fuchshofer et al., 2007; Takahashi et al., 2014). Our results showed that cell senescence and shear stress made no difference to the COLA-1 and COLA-4 mRNA expression of PTM cells under current experimental conditions. These results together suggested that the cell senescence and shear stress altered ECM turnover by regulating MMP expression but not collagen expression. In normal cells, the increased shear stress induced by the elevated IOP (Ethier et al., 2004) led to the up-regulation of expression of MMP, which accelerates ECM degradation and helps to lower the IOP (Turturro et al., 2013; Kennedy et al., 2019). Our results suggest that cell senescence disrupted this feedback, which may eventually contribute to the development of POAG.

To illuminate the mechanism underlying the response of MMPs to the shear stress and the cell senescence, we further studied the expression and phosphorylation of ERK for normal and senescent PTM cells. Our results indicated that the ERK phosphorylation in normal PTM cells was promoted significantly after 12-h shear stress exposure, whereas remarkable suppression of ERK phosphorylation was observed in the senescent PTM cells. This response of ERK phosphorylation to the shear stress and the cell senescence was consistent with that of MMPs, suggesting that the ERK pathway might be involved in modulating MMP expression. In addition, accumulating evidence regarding shear stress-regulated ERK phosphorylation indicates that the initiation and duration of ERK phosphorylation showed cell-type- and stress-type-dependent behavior (Jo et al., 1997; Go et al., 1999; Lee D. Y. et al., 2010; Lee M. Y. et al., 2010; Fukada et al., 2017; Choi et al., 2019; Zhou et al., 2020). For example, Jo et al. found that ERK phosphorylation was up-regulated by laminar shear stress with a maximum at 5 min and a minimum at 30 min in bovine aortic endothelial cells (Jo et al., 1997). Lee et al. observed 2-fold activation of ERK in human osteoblast-like MG63 cells in response to the oscillatory shear stress, as measured from 5 min to 24 h (Lee D. Y. et al., 2010). Our results from short-term exposure to the shear stress indicated that the changes in ERK phosphorylation of the senescent PTM cells might occur at a relatively earlier time point after shearing compared to normal PTM cells.

Additionally, mechanical stress on the outflow pathways oscillates in the eye due to the fluctuation of IOP (Norouzpour and Mehdizadeh, 2012; Zou et al., 2014; Huang et al., 2015; Sherwood et al., 2019; Karimi et al., 2022). Although it is difficult to precisely measure the shear stress on TMCs with the change of IOP, it is commonly believed that the mechanotransduction properties of TM cells regulate the rhythmic IOP fluctuations and control the outflow pathway in response to rapid IOP elevations induced by stressful situations (Johnstone, 2004; Carreon et al., 2017; Turner et al., 2019). The most exciting finding in our study is that senescent PTM cells failed to respond actively to the shear stress. We believe that the senescence-induced impairment of mechanotransduction in TMCs limits the ability to modulate the pulsatile flow of the aqueous fluid while IOP fluctuates, which may eventually lead to dysregulation of IOP and glaucoma.

In conclusion, pTMCs can sense and respond to the shear stress by modifying biomechanical properties and physiological functions. However, cell senescence altered the mechanobiological response and in most cases, rendered the cells less responsive to the shear stress, which may lead to progressive failure of cellular TM function with age. Despite the importance of the mechanobiology of TMCs, our knowledge about TMCs’ behaviors in response to mechanical stress in glaucoma or aging is highly limited. This work gives us new clues to the role of senescence in regulating IOP by affecting TMC dysfunction, which would deepen our understanding of the pathophysiology of POAG.

## Data Availability

The original contributions presented in the study are included in the article/Supplementary Material further inquiries can be directed to the corresponding authors.

## Supplementary material

SUPPLEMENTARY FIGURE S1 Morphology and myocilin (MYOC) gene expression of PTM cells. **(A)** Image of primary PTM cells. **(B)** Image of passage PTM cells. **(C)** Staining for fibronectin (FN) and laminin (LN) of PTM cells. **(D,E)** Myocilin gene expression in PTM cells after corticosteroid treatment. There was a substantial increase in myocilin expression after dexamethasone (DEX) treatment (****p* < 0.001).
