# Supplementary figures and images for: Cell senescence alters responses of porcine trabecular meshwork cells to shear stress
Source: Front Cell Dev Biol. 2022 Nov 21;10:1083130. doi: 10.3389/fcell.2022.1083130 (PMC9721263; doi:10.3389/fcell.2022.1083130)

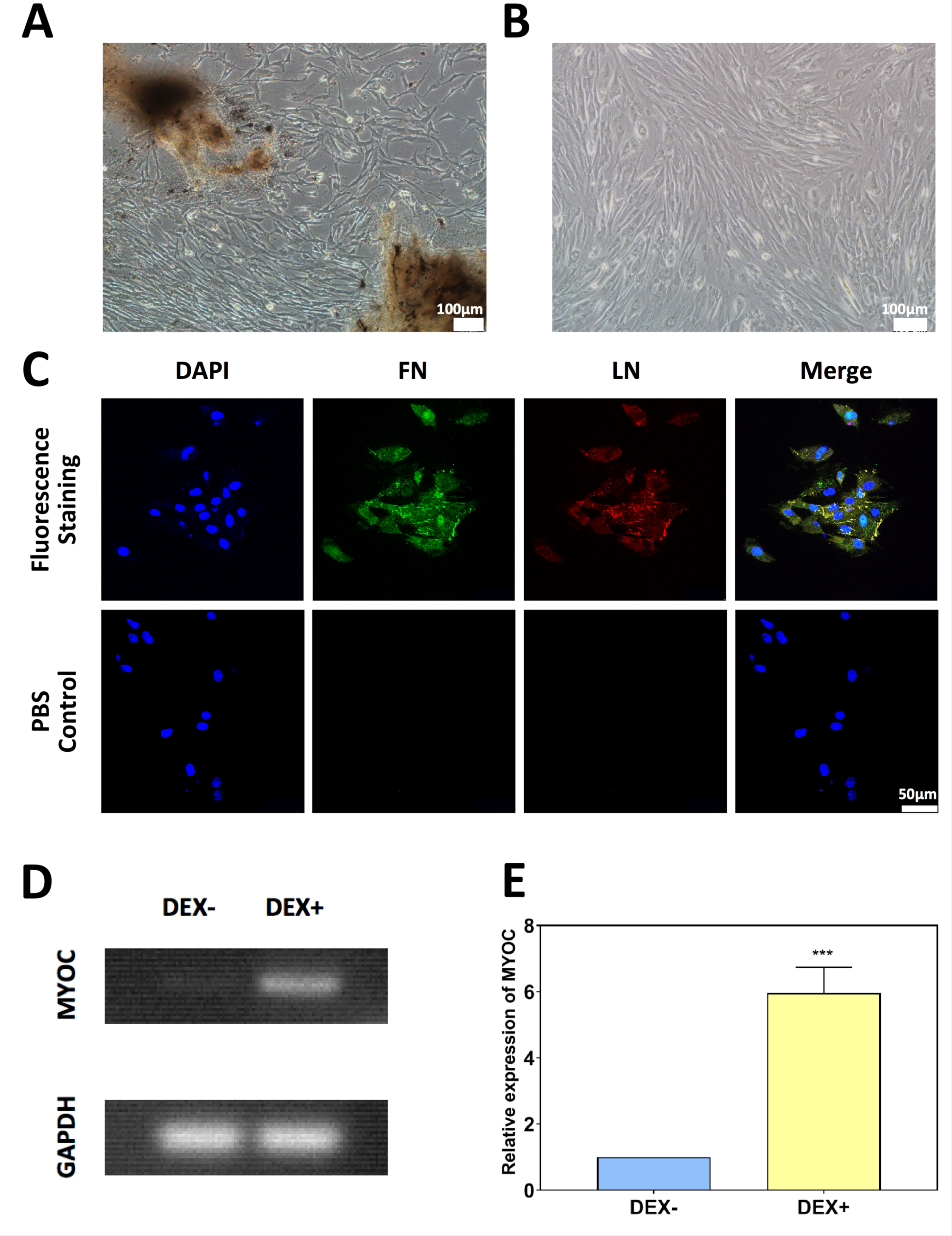

Supplement: Supplementary file 1 [file Image1.TIF]
